# Modeling changes in biomarkers in Gaucher disease patients receiving enzyme replacement therapy using a pathophysiological model

**DOI:** 10.1186/1750-1172-9-95

**Published:** 2014-06-30

**Authors:** Marie Vigan, Jérôme Stirnemann, Catherine Caillaud, Roseline Froissart, Anne Boutten, Bruno Fantin, Nadia Belmatoug, France Mentré

**Affiliations:** 1 INSERM, IAME, UMR 1137, INSERM, F-75018 Paris, France; 2 Univ Paris Diderot, IAME, UMR 1137, Sorbonne Paris Cité, F-75018 Paris, France; 3 Referral Center for Lysosomal Diseases (RCLD), Paris, France; 4 Division of General Internal Medicine, Faculty of Medicine, Geneva University Hospital, Geneva, Switzerland; 5 Laboratoire de Biochimie, Métabolomique et Protéomique, Hôpital Necker-Enfants Malades, AP-HP, Paris, France; 6 Laboratoire des Maladies Héréditaires du Métabolisme, Centre de Biologie Est, Hospices Civils de Lyon, Bron, France; 7 Laboratoire de Biochimie, Hôpital Bichat, Paris, France; 8 Service de Médecine Interne, Hôpital Beaujon, AP–HP, Clichy, France

**Keywords:** Gaucher disease, French registry, Enzyme replacement therapy, Imiglucerase, Glucosylceramide, Ferritin, Chitotriosidase, Hemoglobin, Platelets, Model

## Abstract

**Background:**

Gaucher disease (GD) is a rare recessively inherited disorder caused by deficiency of a lysosomal enzyme, glucocerebrosidase. Accumulation of glucosylceramide or glucosylsphingosine in macrophages leads to increased production of ferritin and chitotriosidase and to decreases in hemoglobin concentration and platelet count, which are used as blood biomarkers. GD is treated by enzyme replacement therapy (ERT) or, sometimes by substrate reduction therapy. However, no physiological model for analysis of biomarkers change during ERT has been proposed. We aimed to develop a pathophysiological model to analyze biomarker’s response to ERT and several covariates impact.

**Methods:**

Changes in blood ferritin, chitotriosidase, hemoglobin and platelets were analyzed in French GD Registry patients receiving imiglucerase/alglucerase as ERT. We used simplified exponential pathophysiological model, with initial concentration, biomarkers amplitude of variation and rate constant of normalization during ERT. Changes in four biomarkers were analyzed separately and then all four together from initiation to discontinuation of ERT, or until the end of follow-up. Several covariates were tested, including age at ERT initiation, splenectomy, sex, genotype (N370S/N370S), and ERT dose.

**Results:**

An exponential model gave a good data fit. The four biomarkers analysis showed that the rate of nomalization was the same for all biomarkers, with a half-life of 0.5 years. Predicted values of biomarkers at ERT’s steady state were 40% and 10% of initial concentrations, for ferritin and chitotriosidase, respectively, and 120% and 200% for hemoglobin and platelets, respectively. We found that 3 covariates had an effect on initial concentration or on amplitude of variation in ferritin, hemoglobin and platelets: women and patients under 15 years of age had lower ferritin and hemoglobin concentrations, and patients under 15 years of age had higher platelet count. Splenectomized patients had higher ferritin concentrations and platelet count and lower amplitude of variation of hemoglobin.

**Conclusion:**

We report the first dynamic model of biomarker changes in GD. It enabled us to estimate that 95% of biomarker response to ERT was achieved in 2 years, but with high inter-patient variability. We also found that with the current treatment, normalization of chitotriosidase and ferritin will occur in about 65% of patients.

## Background

Gaucher disease (GD) [1] is a recessively inherited lysosomal storage disorder caused by deficiency of a lysosomal enzyme, glucocerebrosidase (EC 3.2.1.45), which leads to insufficient clearance of the enzyme’s substrate, cellular glucosylceramide. Pathologic accumulations of glucosylceramide (or other substrates, such as glucosylsphingosine) in the lysosomes of tissue macrophages (Gaucher cells) results in splenomegaly, hepatomegaly and multiple forms of skeletal disease [2]. Three clinical phenotypes have been described: type 1, the prevalent form usually defined by the absence of central nervous system impairment; and types 2 and 3, both rare and severe, have central neurological involvement [3]. GD diagnosis is confirmed by the detection of low glucocerebrosidase activity, usually less than 30% of the normal value in peripheral leukocytes. Genotyping can sometimes provide prognostic information [4]. More than 250 mutations of the GBA1 gene encoding lysosomal glucocerebrosidase have been reported as being associated with GD, but the predominant mutation in type 1 GD is called N370S (or c.1226A > G) [5]. The N370S mutation is usually protective against neuronopathic disease. GD can be treated by enzyme replacement therapy (ERT). The first enzyme preparation used to treat GD consisted of placenta-derived glucocerebrosidase (alglucerase available in 1991, Genzyme Corporation) with modified mannose-terminated glycans, allowing more selective uptake by tissue macrophages, the prominent storage cells in GD [6-8]. This preparation was replaced in 1996 by recombinant enzyme (imiglucerase), which was therapeutically equivalent in terms both of safety and efficacy [9]. Two new biosimilar agents are now available: velaglucerase-alfa (Shire) [10] and taliglucerase-alfa (Pfizer) [11]. ERT reduces macrophagic substrate accumulation, but no routine substrate assay is currently available. A substrate reduction therapy (miglustat, Actelion) that can be prescribed in a few indications has been available since 2002.

The levels of several biomarkers (e.g., in our article, ferritin and chitotriosidase, but also tartrate-resistant acid phosphatase or angiotensin-converting enzyme, not analyzed in this study) change during the clinical course of GD [12-14] due to macrophagic activation: their concentrations rise with disease progression and generally decrease during ERT [12,15,16]. These variations can predict bone complications [17]. High blood ferritin during the course of type 1 GD may reflect macrophage activation triggered by substrate accumulation, demonstrated by increases in CCL18 and macrophage inflammatory protein-1α or 1β [18,19]. ERT is associated with a dramatic decrease of blood ferritin [16,17], which is more pronounced in patients with an intact spleen [20]. Chitotriosidase is massively produced by storage cells and there is a linear relationship between chitotriosidase and glucosylceramide levels, as shown in spleen sections from patients with GD [21]. Chitotriosidase values drop sharply during ERT, when substrate accumulation decreases, coinciding with clinical improvements [12].

Hematological abnormalities (anemia and thrombocytopenia) are common in GD, because Gaucher cell infiltration leads to hypersplenism (increased destruction or sequestration of red blood cells or platelets) and bone-marrow insufficiency (decreased production) [22]. Splenectomy, performed essentially before 1991, increases baseline platelet count and decreases the slope of platelet clearance during ERT [17]. Anemia and thrombocytopenia can be used as biomarkers to manage GD patients.

Grabowski et al. [23] developed an Emax model to describe changes in hemoglobin and platelets and in splenic and hepatic volume during ERT of patients in the International Collaborative Gaucher Group Registry. Biomarkers of French GD patients from a single center was modeled before and during ERT by Stirnemann et al. [17], but no physiological model was proposed to analyze changes in biomarkers levels during ERT. Nonlinear mixed effects models [24] are widely used to analyze biological processes described by repeated longitudinal data. They allow estimation of the mean value of the parameters and their inter-individual variability. These models allow a sparse sampling design with few data points per individual in a large set of individuals.

The aim of this study was to develop a pathophysiological model explaining the response of biomarkers (ferritin, chitotriosidase, hemoglobin, platelets) to ERT and to analyze the influence of several covariates.

## Patients and methods

### Patients and data

We analyzed changes in four biomarkers, ferritin, chitotriosidase, hemoglobin and platelets, in patients receiving ERT with alglucerase from 1991 to 1996 and thereafter imiglucerase, from the French GD Registry (FGDR) [25]. The French Data Protection Commission (CNIL) approval of the FGDR required oral or written informed consent from patients or their parents. The local Institutional Review Board of Northern Paris Hospitals, Paris–Diderot University, AP–HP (Ethics Committee) reviewed and approved the initial research project (220–08). These patients were included in the study if the last follow-up visit was between 2009 and 2010. This investigation was undertaken to describe changes in biomarkers from initiation of ERT until 31 December 2010 corresponding to the limit of collected retrospective data in the FGDR. These data correspond also to those used in the first description of patients of the FGDR [25]. All measurements of biomarkers from 2 years before the initiation of ERT to discontinuation of ERT (interruption of more than 6 months) or the end of follow-up (December 2010) were used. When the chitotriosidase activity was undetectable (patients with probable homozygous chitotriosidase-gene deficiency), this biomarker was not retested [26] and the data were not included in the analysis. When ERT was interrupted for less than 6 months, patients were considered to be still under treatment. Several covariates were tested including age at initiation of ERT, splenectomy, genotype (N370S/N370S or others), sex, dose at initiation of treatment (divided into 3 classes: lower than 90 IU/kg/month, between 90 and 120 IU/kg/month or higher than 120 IU/kg/month) and average dose in the third year of treatment (divided into the same 3 classes) for patients followed up for at least 3 years.

### Model for biomarker changes during ERT

**Figure 1:**
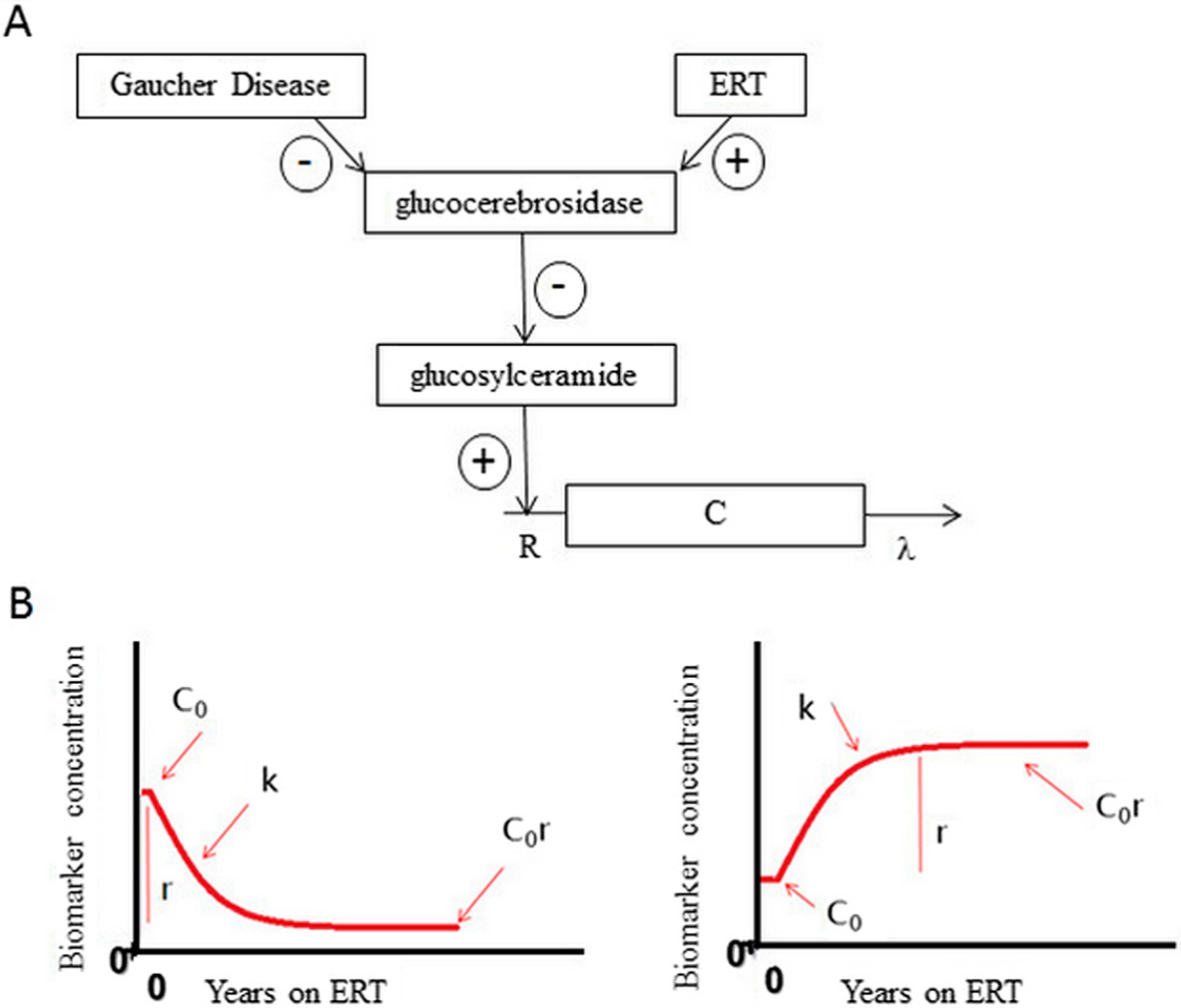
**Scheme of the effect of Gaucher disease on biomarkers and the effect of ERT. A**: Mechanistic pathophysiological model. Plus sign (+) indicates stimulation and minus sign (−) indicates inhibition. C is chitotriosidase produced at a rate of R and removed at a rate of λ **B**: Simplified model of changes in biomarkers during ERT. Left: biomarkers that decrease during ERT (ferritin, chitotriosidase); right: biomarkers that increase during ERT (hemoglobin, platelets). *C*_0_ is initial concentration, *k* rate of decrease (or increase) and *C*_0_*r* estimated corrected value at steady state of ERT.

We defined a pathophysiological model [27] of GD and treatment by ERT (Figure 1). GD is caused by a glucocerebrosidase deficiency that leads to an accumulation of glucosylceramide, which is no longer degraded. As a consequence, there is an increase in ferritin and chitotriosidase production. With ERT, patients are supplied with glucocerebrosidase which leads to the degradation of the glucosylceramide and then improvements of biomarkers levels. The differential equation of this model for chitotriosidase is presented in Supplementary material (Additional file 1). As only biomarker measurements were available, we made a further simplification. Since the rate constant of biomarker elimination is very rapid compared with the rate constant of normalization of glucosylceramide under treatment (k), it was neglected so that we have an exponential increase for chitotriosidase. For chitotriosidase activity, the model is:

$$C\left(t)=C_{0}[r+\left(1-r\right)\exp\left(-\mathrm{kt}\right)\right)]$$

where *C*_0_ is the initial concentration, r is the amplitude of variation and k is the rate constant of normalization during ERT. For serum ferritin, a similar reasoning leads to the same equation. From the model, we can derive the corrected values of biomarkers at steady state of ERT as C_0_r (Figure 1) and the normalization half-life as $\;\frac{\log\left(2\right)}{k}$.

Even though two different mechanisms explain hematological abnormalities (destruction by hypersplenism and decrease hematopoiesis), we proposed to simplify the model with only one predominant mechanism (destruction). By similar reasoning, for hemoglobin and platelets, the model is:

$$C\left(t\right)=C_{0}\left[1+\left(r-1\right)\left(1-\exp\left(-\mathrm{kt}\right)\right)\right]$$

which increases from *C*_0_ to *C*_0_*r* (Figure 1).

### Statistical analyses

We used a nonlinear mixed effects model to analyze measurements of the four biomarkers. First, we performed a separate analysis of each biomarker. We assumed an exponential random effect on all parameters and that these random effects have a normal distribution with a mean of 0 and a standard deviation of ω. The residual error model was supposed to be proportional with a standard deviation, σ. We also tested the correlation between random effects. Model selection was performed using the Bayesian information criterion (BIC) which adds to the log-likelihood penalty increases with the number of model parameters and the sample size used (BIC = −2 × logL + k log (N)), where L is the maximum likelihood, k is the number of model parameters and N is the number of patients. BIC is a parsimonious criterion where the lowest value corresponds to the best model. We compared our exponential model with the Emax model of Grabowski et al. [23] using the BIC.

We tested the impact of the covariates mentioned above to explain part of the variability [28]. As only discrete covariates were considered, the effect of the covariate is to change the parameter by exp(β) where β is the estimated regression coefficient. The final model was built using a two-step approach. In the first step, individual empirical Bayesian estimates of parameters were generated from the basic model without covariates. We searched for univariate associations with covariates using Wilcoxon or Spearman tests. In the second step, all covariates with a p-value of < 0.2 were entered into a multivariate model. We then performed a multivariate analysis using a forward selection. P-values of the Wald test were assessed at the 0.05 level. Splenectomy has an impact on changes in platelet count [17]. So we considered (and tested) that splenectomy has an impact on the initial platelet count and on its amplitude of variation.

We then analyzed the 4 biomarkers jointly and evaluated whether one or more parameters were correlated or if several parameters had similar values, to simplify the model. We evaluated the final model with various goodness-of-fit plots [29]: individual fits, graphs of individual weighted residuals versus time and visual predictive check. The significant covariates obtained previously in models of each biomarker were added. Then, we performed a backward selection and computed the P-values of the Wald test. Following estimation of population parameters, we could estimate individual parameters. Individual empirical Bayesian estimates were obtained as maximum a posteriori. From the individual parameters, we estimated individual half-life, time to 95% of response (as 4.3 half-lives), values of biomarkers at steady state of treatment and whether this value is normal or not.

Estimations were performed using the Stochastic Approximation Expectation Maximization (SAEM) algorithm in MONOLIX 4.2.0 (Lixoft, Orsay, France, available at http://www.lixoft.com) [30]. BIC and log-likelihood were estimated by importance sampling.

## Results

### Patient characteristics

In the FGDR, 233 patients receiving imiglucerase had a medical visit in 2009 or 2010. The diagnosis of GD was confirmed biochemically in all patients, demonstrating a deficiency of glucocerebrosidase activity either in leukocytes or in a fibroblast cell line. Their median (range) follow-up with ERT was 9 (0–19) years. Median age at the initiation of ERT was 34.3 (1.0-76.0) years, with 18% < 15 years of age, 115 (49.4%) were male, and 61 (26.2%) had a splenectomy before the initiation of the ERT. Twenty-six (11.1%) were N370S/N370S homozygotes. Other characteristics of the patients are presented in Table 1. The median (range) dose at initiation of ERT was 120 IU/kg/month (28 IU/kg/month - 240 IU/kg/month) with 169 (79.0%) patients with 120 IU/kg/month. The average dose during the third year was lower than 90 IU/kg/month for 40 (17.4%) patients, higher than 120 IU/kg/month for 13 (5.7%) and between 90 and 120 IU/kg/month for 176 (76.9%). In some patients there was no monitoring of biochemical parameters during follow-up, or monitoring of only one of the four biomarkers. Therefore, data were available for 129, 142, 191 and 191 patients with a total number of observations of 586, 596, 1287, and 1336 for ferritin, chitotriosidase, hemoglobin and platelets, respectively. Median length of follow-up during ERT was 5 years for ferritin and 6 years for the other biomarkers. Over 65% of the patients were followed up for more than 3 years.

**Table 1:** Patient characteristics

		No.*	**Value**
Sex, n (%)		233	
	Female		118 (50.6)
	Male		115 (49.4)
Age, years, median (range) [IQR]			
	Diagnosis	233	14.5 (0.5-67.5) [8.4;34.0]
	Initiation of ERT	233	34.3 (1.0-76.0) [20.6;48.0]
Patients <15 at diagnosis, n (%)		233	87 (62.7)
Patients <15 at initiation of ERT, n (%)		233	41 (17.6)
Type, n (%)		233	
	1		226 (97.0)
	3		7 (3.0)
Genotype, n (%)		194	
	N370S/N370S		26 (13.4)
	N370S/L444P		31 (15.9)
	L444P/L444P		4 (2.1)
	N370S/Other		100 (51.5)
	L444P/Other		10 (5.2)
	Other/Other		23 (11.9)
Initial test leading to GD diagnosis†, n (%)**		153	
	Enzyme assay		34 (22.2)
	*GBA*-gene sequencing		1 (0.7)
	Bone-marrow aspiration		79 (51.6)
	Bone-marrow biopsy		13 (8.5)
	Bone biopsy		5 (3.3)
	Liver biopsy		3 (1.9)
	Spleen histology		17 (11.1)
	Other		1 (0.7)
Splenectomy at initiation of ERT, n (%)		233	61 (26.2)
Patients with bone events, n (%)			
	At initiation of ERT	233	65 (27.9)
	On ERT	233	33 (14.1)
Initial dose, n (%)		213	
	< 90 IU/kg/month		28 (13.1)
	90- 120 IU/kg/month		174 (81.7)
	> 120 IU/kg/month		11 (5.2)

*GBA* = glucosidase-β acid. *No. represents the number of patients with available information.

† Several symptoms for each patient. **All patients had diagnosis confirmed by enzymatic assay.

### Changes in biomarkers

**Figure 2:**
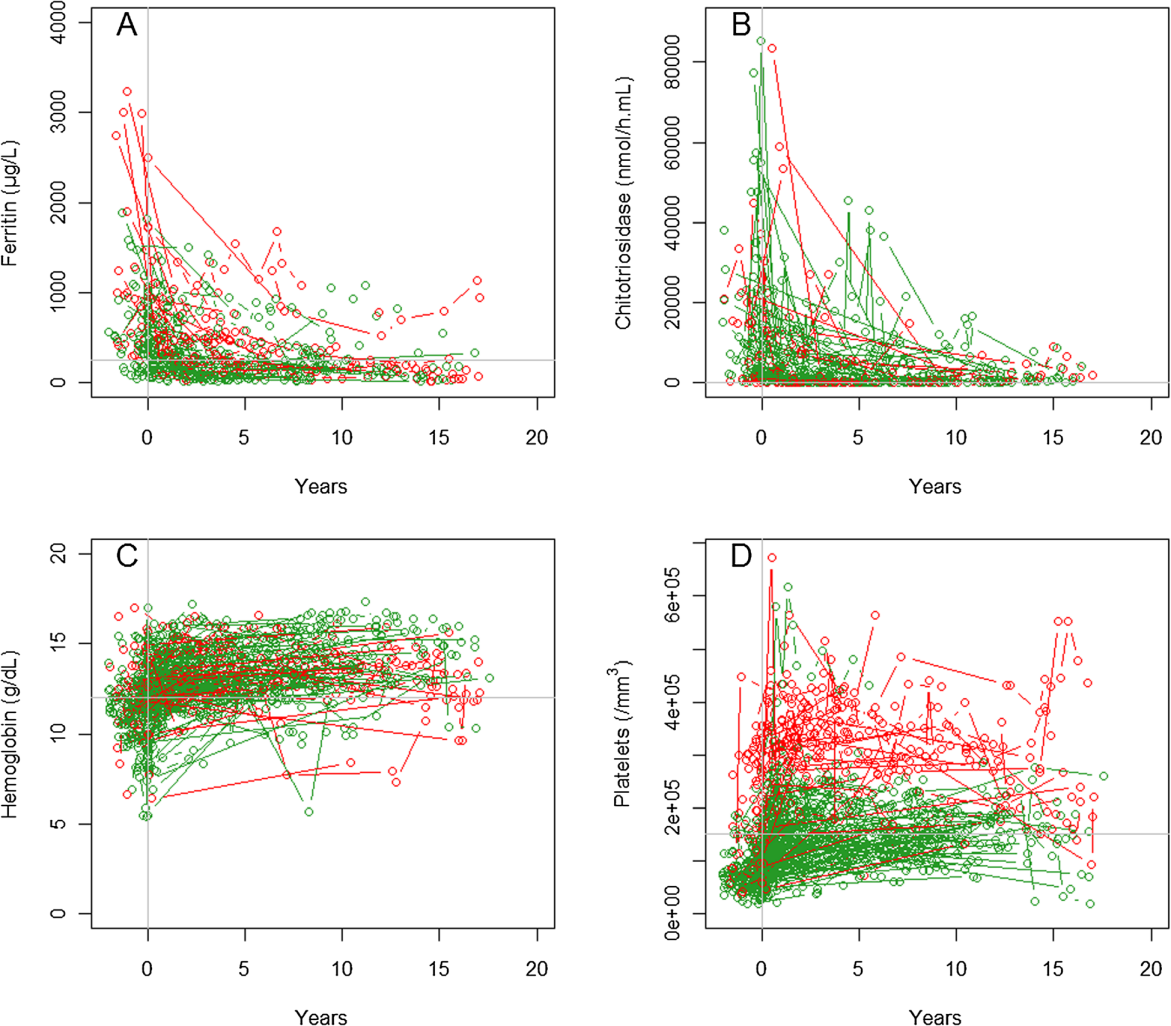
**Spaghetti plot of biomarker concentration versus time during ERT for A) ferritin, B) chitotriosidase, C) hemoglobin and D) platelets.** Time 0 corresponds to initiation of ERT, vertical gray line. Green lines correspond to non-splenectomized patients and red lines to splenectomized patients. Gray horizontal lines correspond to the limit of usual values: ferritin <250 μg/L, chitotriosidase <100 nmol/mL/h, hemoglobin >12 g/dL, platelet count >150 000/mm^3^.

Figure 2 shows the individual changes in each biomarker during ERT in a spaghetti plot. The exponential model fitted the data. Comparison of our exponential model with the Emax model gave a gain BIC of 3 for ferritin, 12 for chitotriosidase, and 22 for platelets for our model and a loss of 7 for hemoglobin. We tested the impact of covariates in the model of separate biomarkers and found that 3 covariates (initiation of ERT < 15 years of age, splenectomy and sex) had an effect on the initial concentration and/or on the amplitude of variation in ferritin, hemoglobin and platelets. Genotype and dose had no significant impact on the parameters and none of the covariates had a significant impact on chitotriosidase.

**Figure 3:**
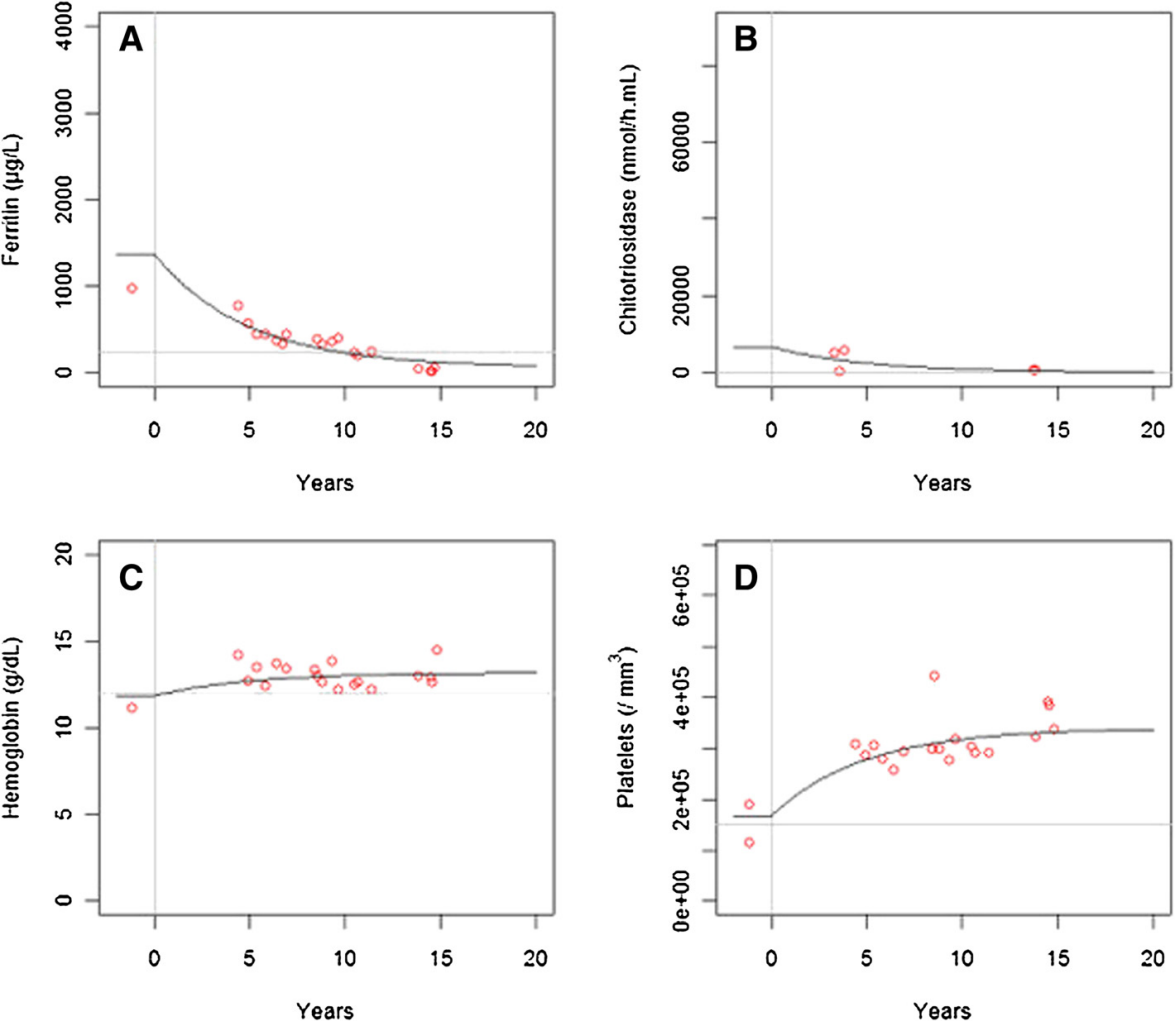
**Individual fits for two patients of biomarkers versus time during ERT for A) ferritin, B) chitotriosidase, C) hemoglobin and D) platelets for two patients.** Top: splenectomized patient with red dots. Bottom: non-splenectomized patient with green dots. The black line corresponds to the curve predicted by the model. Time 0 corresponds to initiation of ERT, vertical gray line. Gray horizontal lines correspond to the limit of usual values: ferritin <250 μg/L, chitotriosidase <100 nmol/mL/h, hemoglobin >12 g/dL, platelet count >150 000/mm^3^.

The best model of analysis of the four biomarkers together was the one with the same parameter k for all biomarkers, so we considered a rate constant of normalization during ERT common to the four biomarkers. The model with the same parameter k had a smaller BIC of 22 than the model with 4 different values for parameters k. Values of parameters without covariates (only splenectomy for platelets) are given in Table 2 and Table 3: Predicted values of biomarkers at steady state of ERT were 38% and 10% of initial concentrations, for ferritin and chitotriosidase, respectively, and 117% and 200% for hemoglobin and platelets, respectively. Estimated corrected values at steady state of ERT were 179.4 ng/L, 792 nmol/h.mL and 13.9 g/dL for ferritin, chitotriosidase and hemoglobin, respectively. The estimated corrected platelet counts at steady state of ERT were 156,030/mm^3^ and 195,037/mm^3^ for non-splenectomized and splenectomized patients, respectively. Individual predicted values show that, under current ERT, normalization will occur for 58% of the patients for ferritin, 66% for chitotriosidase, 88% for hemoglobin and 64% for platelets. Figure 3 shows individual fits for one splenectomized patient and one non-splenectomized. Relative standard errors were lower than 20% for both fixed effects and variabilities. Diagnostic plots (Additional file 2) show that the model describes the data adequately.

**Table 2:** Values of fixed effects and variabilities for the final model of 4 biomarkers without covariates

**Fixed effects**	**Estimates**	**RSE (%)**	**P-value**
C_0F_ ( μg/L)	598	8	
C_0C_ (nmol/h.mL)	7920	11	
C_0H_ (g/dL)	11.6	1	
C_0P_ (/mm^3^)	74300	4	
exp(β_ splen)	2.5	8	< 10^−10^
r_F_	0.4	8	
r_C_	0.1	14	
r_H_	1.2	1	
r_P_	2.1	4	
exp(β_ splen)	0.5	19	10^−7^
k (years^−1^)	1.4	10	
**Variabilities**			
ω___ C_0F_	83	8	
ω_C_0C_	120	7	
ω___ C_0H_	13	6	
ω_C_0P_	42	6	
ω_r_F_	67	9	
ω_r_C_	117	10	
ω_r_H_	10	10	
ω_r_P_	31	10	
ω_k	90	10	
σ_F	30	4	
σ_C	61	4	
σ_H	7	2	
σ_P	20	3	

C_0_ is initial concentration, r amplitude of variation and k the rate of normalization during ERT. F corresponds to ferritin, C to chitotriosidase, H to hemoglobin and P to platelets. The P-value is for the Wald test. Exp β represents the effect of the covariates. Splen corresponds to the covariate splenectomy. ω: standard deviation of inter-individual variability, σ: standard deviation of proportional residual error. RSE: relative standard error. For example, baseline ferritin concentration is C_0F_ =598 μg/L, and the estimated corrected value at steady state of ERT is *C*_0*F*_ × *r*_*F*_ =  179.4 μg/L. Baseline platelet count in non-splenectomized patients is *C*_0*P*_ =  74,300/mm^3^ and C_0*P*_ exp(β _ splen) = 185,750 /mm^3^ in splenectomized patients. The estimated corrected platelet count at steady state of ERT in splenectomized patients is (*C*_0*P*_ × exp(*β* _ *splen*)) × (*r*_0_ × exp(*β* _ *splen*)) = 195,037 /mm^3^.

**Table 3:** Estimated individual characteristics of biomarker response to ERT for the 197 patients

	**Median (range) at steady state**	**Normalization n (%)**
Half-life of response (years)	0.5 (0.01-3.1)	
Time to 95% of response (years)	2.1 (0.4-13.4)	
Ratio (level at steady state/level at ERT initiation)		
Ferritin (%)	38.1 (2.1-101.5)	
Chitotriosidase (%)	10.1 (0.8-116.7)	
Hemoglobin (%)	117.3 (88.6-173.1)	
Platelets (%)	200.4 (83.5-437.8)	
Predicted value at steady state		
Ferritin (μg/L)	202.0 (16.5-1280.1)	115 (58.4)
Chitotriosidase (nmol/h.mL)	820 (8–18183)	131 (66.5)
Hemoglobin (g/dL)	13.5 (8.1-17.2)	174 (88.3)
Platelets (/mm^3^)	172 000 (48 000–526 000)	126 (64.0)

Normalization was defined by: ferritin <250 μg/L, chitotriosidase <100 nmol/mL/h, hemoglobin >12 g/dL, platelet count >150 000/mm^3^.

**Figure 4:**
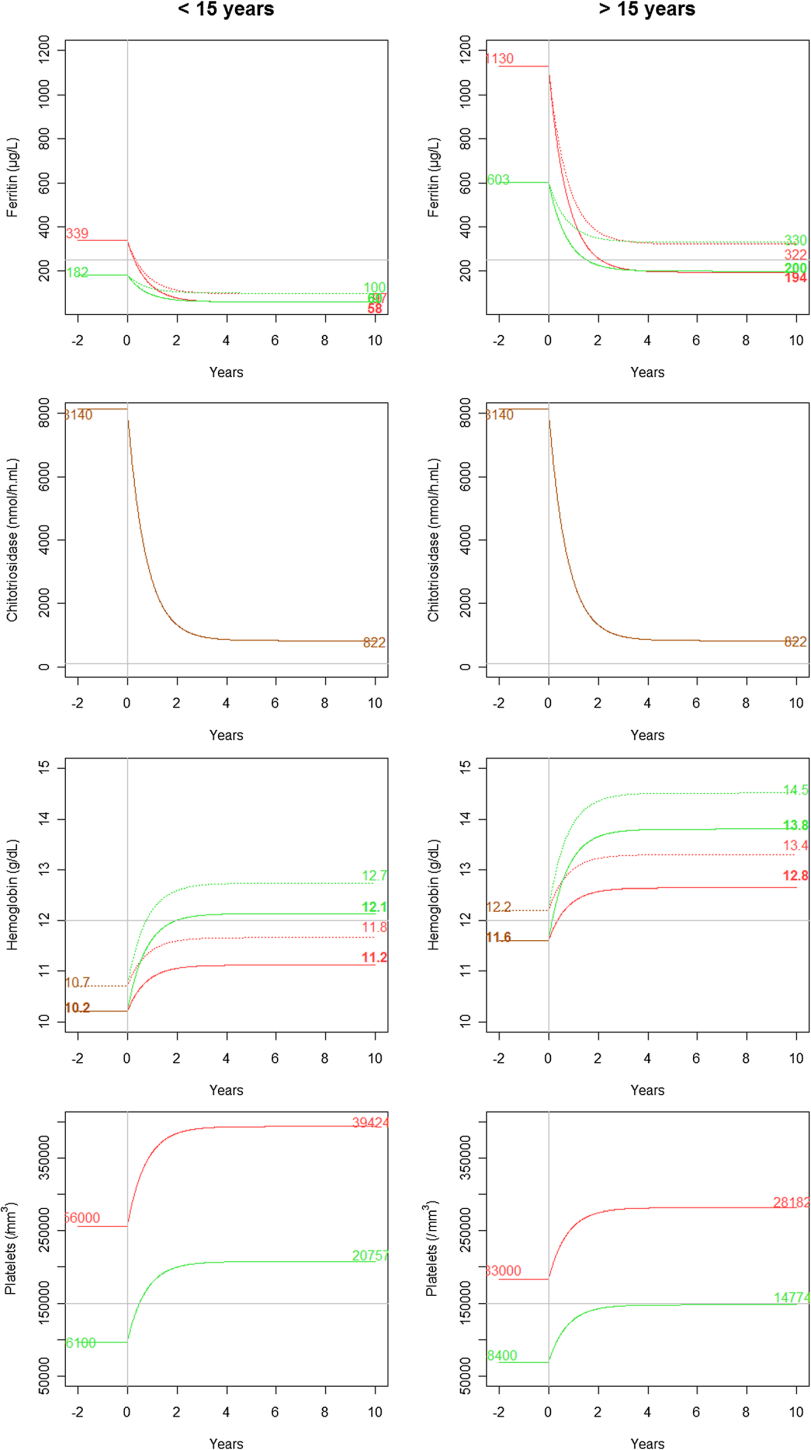
**Biomarker responses for a median patient for the final model according to significant covariates for ferritin, chitotriosidase, hemoglobin and platelets.** A green line is for non-splenectomized patients and a red line for splenectomized patients. A dotted line is for man and continuous for woman. For platelets, no distinction between man and woman and change is a continuous line. Initial concentration and concentrations at steady state of ERT are indicated, in green for non-splenectomized, red for splenectomized patients, and in orange when no distinction can be made depending on splenectomy. Lines are thin for man and bold for woman. Left: patients <15 years of age. Right: patients > 15 years of age.

Values of parameters in the joint model with covariates are given in Table 4. The covariate effects were the same as in the models considering the biomarkers separately. Figure 4 shows the response of the biomarkers to ERT, according to significant covariates. Non-splenectomized (p-value = 10^−6^) and age <15 years (p-value < 10^−10^) patients had a lower initial concentration of ferritin. Women (p-value = 0.002) and non-splenectomized (p-value = 10^−4^) patients had a lower amplitude of variation in ferritin. Women (p-value = 0.01) and age <15 years (p-value = 10^−8^) patients had a lower initial concentrations of hemoglobin. Splenectomized (p-value = 10^−5^) patients had a lower amplitude of variation in hemoglobin. Splenectomized (p-value < 10^−10^) and age <15 years (p-value = 10^−6^) patients had a higher initial platelet count. Splenectomized (p-value = 10^−8^) patients had a lower amplitude of variation in platelets.

**Table 4:** Values of fixed effects and variabilities for the final model with significant covariates

**Fixed effects**	**Estimates**	**RSE (%)**	**P-value**
C_0F_ ( μg/L)	603	9	
exp(β_ splen)	1.9	24	10^−6^
exp(β_ < 15)	0.3	14	< 10^−10^
C_0C_ (nmol/h.mL)	8140	12	
C_0H_ (g/dL)	11.6	1	
exp(β_ man)	1.1	40	0.01
exp(β_ < 15)	0.9	19	10^−8^
C_0P_ (/mm^3^)	68400	4	
exp(β_ splen)	2.7	8	< 10^−10^
exp(β_ < 15)	1.4	24	10^−6^
r_F_	0.3	13	
exp(β_ splen)	0.5	29	0.0005
exp(β_ man)	1.7	32	0.002
r_C_	0.1	14	
r_H_	1.2	1	
exp(β_ splen)	0.9	25	10^−5^
r_P_	2.2	4	
exp(β_ splen)	0.7	19	10^−8^
k (years^−1^)	1.4	11	
**Variabilities**			
ω___ C_0F_	63	8	
ω_C_0C_	122	7	
ω___ C_0H_	12	6	
ω_C_0P_	40	6	
ω_r_F_	70	10	
ω_r_C_	113	10	
ω_r_H_	9	9	
ω_r_P_	31	9	
ω_k	97	10	
σ_F	29	4	
σ_C	61	4	
σ_H	7	2	
σ_P	20	2	

C_0_ is initial concentration, r amplitude of variation and k the rate of normalization during ERT. F corresponds to ferritin, C to chitotriosidase, H to hemoglobin and P to platelets. The P-value is for the Wald test. exp β represents the effect of the covariates. Splen corresponds to the covariates splenectomy, <15 indicates patients under 15 years of age and man indicates the covariate sex. ω: standard deviation of inter-individual variability, σ: standard deviation of proportional residual error. RSE: relative standard error.

In the final model, we estimated that during ERT the half-life of normalization was 0.5 years (95% CI = [0.4-0.6]), with a variability of 97%. Half-life was 0.5 (0.1-3.1) years and 95% of response was obtained after 2 (0.4-13.3) years under ERT. Variabilities were reasonable for ferritin, hemoglobin and platelet count. Greater variability was found for chitotriosidase, doubtless because of variability in measurement error (a sample dilution is usually needed when values are high).

## Discussion

Our pathophysiological model predicts changes in biomarkers on ERT and estimates the rate constant of normalization. For the final model, we estimated a normalization half-life of 0.5 years during ERT for all four biomarkers. Only 2 studies have modeled the changes in biomarkers and they used different models: Emax and linear mixed models. In the study of Grabowski et al. [23], the ERT dose effect had a significant impact on biomarkers with a large sample size. Emax is an empirical model with a hyperbolic function; it is not the result of a physiological model. These models are used in pharmacometrics [27] to study the link between dose and concentration. However, the drug often acts on a biological quantity, modifying its production or elimination. We modeled this quantity by a model with a production rate and a rate constant of elimination. Using physiological knowledge, we obtained differential equations to explain changes in biomarkers over time. For instance, 90% response is obtained after 9 T_50_ for an Emax model, whereas it is reached after only 3.3 half-lives (similar to T_50_) for an exponential model.

Our results show improvement in all biomarkers under ERT (decrease in ferritin and chitotriosidase and increase in hemoglobin and platelets). Stein et al. [20] also highlighted an increase in ferritin and a renormalization under ERT. Hollak et al. [31] reported a decrease of chitotriosidase of 32% in 1 year, and we found a 95% response in 2 years and a 36% response in 1 year. De Fost et al. [32] reported that 53% of patients had anemia at baseline and 58% had thrombocytopenia, with renormalization under ERT, and showed a similar pattern of response after 1 year under ERT.

Patients <15 years of age have lower initial concentrations of ferritin and hemoglobin but higher platelet counts; at initiation of ERT, women have a lower concentration of hemoglobin and splenectomized patients have a higher platelet count and ferritin. For GD children from the International Collaborative Gaucher Group Registry, Kaplan P et al. [33] noted that 50% had platelet counts less than 120 × 10^3^/mm^3^ and 40% had anemia at the time of diagnosis. Hemoglobin and ferritin tend to be lower in GD women, as for other women, probably because of menstruation, with no link to GD. In our model, no covariates had a significant impact on the chitotriosidase changes. In contrast, Stirnemann et al. [17] found that splenectomy and GD genotype affected chitotriosidase activity, but their study was limited by a small number of samples, and they used a different model.

Our model allows estimation of the rate constant of biomarker improvement using a pathophysiological model. Bone events are the most debilitating and disabling complication of GD. With substrate overload, Gaucher cells activate and induce proinflammatory cytokine synthesis which can modify the activity of the osteoblast-osteoclast system and promote lytic phenomena and intraosseous vascular complications [34,35]. Further analysis of the interaction between biomarkers and bone events is needed.

Recently, plasma glucosylsphingosine has been proposed as a biomarker for GD [36,37] and could be used as a reflection of intracellular glucosylceramide. Our model may also predict changes in intracellular glucosylceramide and/or glucosylsphingosine.

Our model could be used to study the effect of biomarker changes on complications such as the occurrence of bone events. Using a few measurements of biomarkers post-treatment, we can estimate the rate constant of normalization and individual amplitude of variation in biomarkers in order to predict further response to treatment. Our model could help to define an individual risk for complications and to refine the best ERT regimens.

Parameters of this pathological model could be used to predict value at steady state with the dosages in the first months of ERT. These predicted values could be used as an individual objective of treatment to modify dosage for each patient. Clinical studies are needed to confirm this hypothesis.

We developed a model which could in the future be used to manage patients, but further studies are needed to confirm clinical applications. This model with 2 parameters (in addition to the baseline value) predicts the level of improvement of biomarkers and the time to 95% response, using only a few measurements per patient.

## Conclusion

This is to our knowledge the first study of changes in biomarkers in Gaucher disease using a pathophysiological model. With the model we estimate that 95% of biomarker response to ERT is achieved in 2 years, but with high inter-patient variability. We also found that with the current treatment, normalization of chitotriosidase and ferritin will occur in about 65% of patients.

## Abbreviations

BIC: Bayesian information criterion; ERT: Enzyme-replacement therapy; FGDR: French Gaucher disease registry; GD: Gaucher disease.

## Competing interests

FM had consulting fees and INSERM UMR1137 received a grant from Sanofi. JS had travel fees from Sanofi-Genzyme. NB had travel fees, consulting fees, fees for speaking and received grants (Genzyme, Shire, Actelion, Pfizer) donated to the Department of Clinical Research of the Assistance-Publique Hôpitaux de Paris. The other authors declared no financial disclosures.

## Authors’ contributions

MV, JS and FM designed the research and analyzed and interpreted the data. MV, JS and FM wrote the first draft of the paper, which was then corrected and approved by all authors.

## Supplementary Material

Additional file 1 Equation describing change in chitotriosidase level. Click here for file

Additional file 2 Goodness-of-fit plots. Click here for file
