# Supplementary material for: Modeling changes in biomarkers in Gaucher disease patients receiving enzyme replacement therapy using a pathophysiological model
Source: Orphanet J Rare Dis. 2014 Jun 30;9:95. doi: 10.1186/1750-1172-9-95 (PMC4094900; doi:10.1186/1750-1172-9-95)
Supplement: Additional file 1 — Equation describing change in chitotriosidase level. [file 1750-1172-9-95-S1.docx]

**Additional file 1: Equation describing change in chitotriosidase level**

As shown in Figure 1, production of glucosyleceramide decreases during ERT. We assume that the baseline value before treatment is $R_{0}$and that it decreases to a value$R_{T}$ with a rate constant k. Therefore, we modeled$R\left( t \right)$ as:

$$R\left( t \right)=R_{T}+\left( R_{0}-R_{T} \right) exp(-kt)$$

The concentration of chitotriosidase can be modeled by the following differential equation:

$$\frac{dC}{dt}(t)=R(t)-\lambda C(t)$$

where $\lambda$is the rate constant of chitotriosidase elimination

The initial condition in the absence of ERT is $C_{0}=\frac{R_{0}}{\lambda}.$ The analytical solution of this equation is given by:

$$C\left( t \right)=\frac{R_{T}}{\lambda}+\frac{R_{0}-R_{T}}{\lambda-k}exp\left( -kt \right)+{(R}_{0}-R_{T})(\frac{1}{\lambda}-\frac{1}{\lambda-k})exp(-\lambda t)$$

We consider that the half-life of chitotriosidase is very short compared with the treatment effect, *i.e* $\lambda\gg k$. Therefore $C\left( t \right)$ can be approximated by:

$$C\left( t \right)\simeq\frac{R_{T}}{k}+\frac{R_{0}-R_{T}}{k}exp\left( -kt \right)$$

We define $C_{T}=\frac{R_{t}}{\lambda}$, corresponding to the concentration at steady state of ERT for chitotriosidase. Then

$$C\left( t \right)=C_{T}+(C_{0}-C_{T})exp\left( -kt \right)$$

We define$r=\frac{C_{T}}{C_{0}}$, corresponding to the amplitude of variation in chitotriosidase, then:

$$C\left( t)=C_{0}[r+\left( 1-r \right)\exp\left( -kt \right) \right)]$$
