# Supplementary material for: Modeling changes in biomarkers in Gaucher disease patients receiving enzyme replacement therapy using a pathophysiological model
Source: Orphanet J Rare Dis. 2014 Jun 30;9:95. doi: 10.1186/1750-1172-9-95 (PMC4094900; doi:10.1186/1750-1172-9-95)

**Additional file 2: Goodness-of-fit plots**

**Figure S1: Visual predictive check of biomarker models for A) ferritin, B) chitotriosidase, C) hemoglobin and D) platelets.** The green lines represent the 10^th^; 50^th^ and 90th percentiles for observed data. The shaded blue and pink areas represent 90% prediction intervals for the corresponding percentiles calculated from simulated data.

**
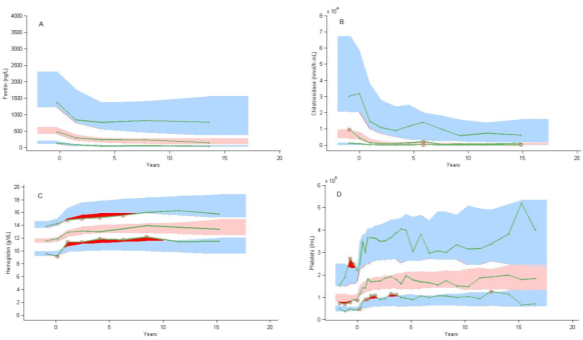
**

**Figure S2: Individuals weighted residuals of the biomarker models versus time for A) ferritin, B) chitotriosidase, C) hemoglobin and D) platelets.** It is expected that residuals do not follow the trend and are distributed around the line y = 0.
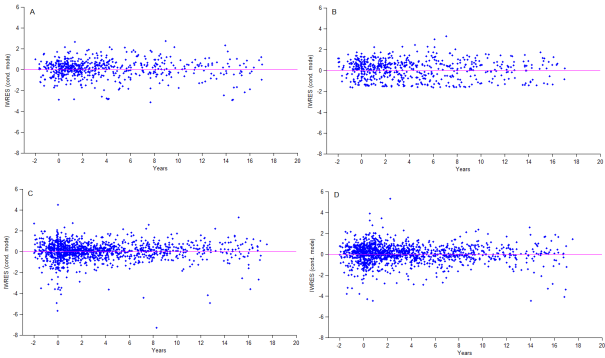

Supplement: Additional file 2 — Goodness-of-fit plots. [file 1750-1172-9-95-S2.docx]
